# *trans*-Di­aqua­bis­(pyridin-2-yl thio­phen-2-yl ketone-κ^2^*N*,*O*)cobalt(II) bis­(tetra­fluorido­borate)

**DOI:** 10.1107/S2414314626007492

**Published:** 2026-07-23

**Authors:** Barry L. Westcott

**Affiliations:** a Department of Chemistry and Biochemistry Central Connecticut State University, 1615 Stanley St New Britain CT 06050 USA; Vienna University of Technology, Austria

**Keywords:** crystal structure, cobalt, pyridyl ketone, thio­phene, twin structure, disorder

## Abstract

The title salt, [Co(C_10_H_7_NOS)_2_(H_2_O)_2_](BF_4_)_2_, crystallizes in the monoclinic system (space group *P*2_1_/*n*). In the complex cation, the central Co^II^ atom (site symmetry 1) has a pseudo-octa­hedral coordination environment with bonding to pyridyl N [2.0189 (13) Å] and carbonyl O [2.0869 (11) Å] atoms from the 2-thienyl 2-pyridyl ketone ligands. The remaining bonds are to water mol­ecules [2.0903 (13) Å], which form a hydrogen-bonded extended structure with BF_4_^−^ counter-ions. The thienyl ring shows disorder through an approximate twofold rotation with an occupancy ratio of 0.951 (3):0.049 (3).

## Abstract 2

A complex of Co^II^ with the ligand 2-thienyl-2-pyridyl ketone shows pseudo-octa­hedral binding of the central metal cation with two ligands bound through the carbonyl oxygen and pyridyl nitro­gen, and two coordinating water mol­ecules in the axial positions.

## Graphical abstract

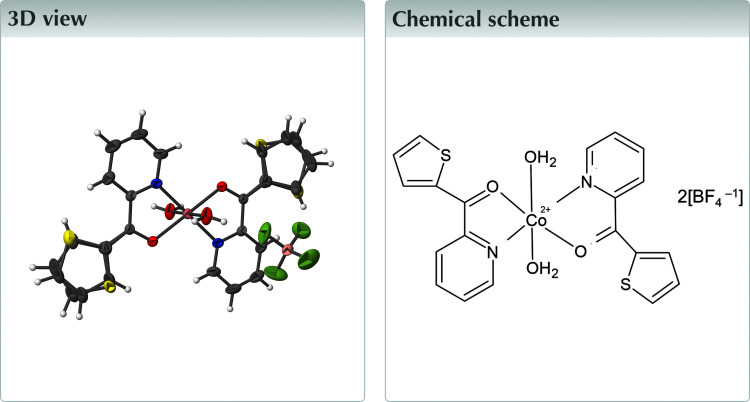

## Structure description

**Figure 1:**
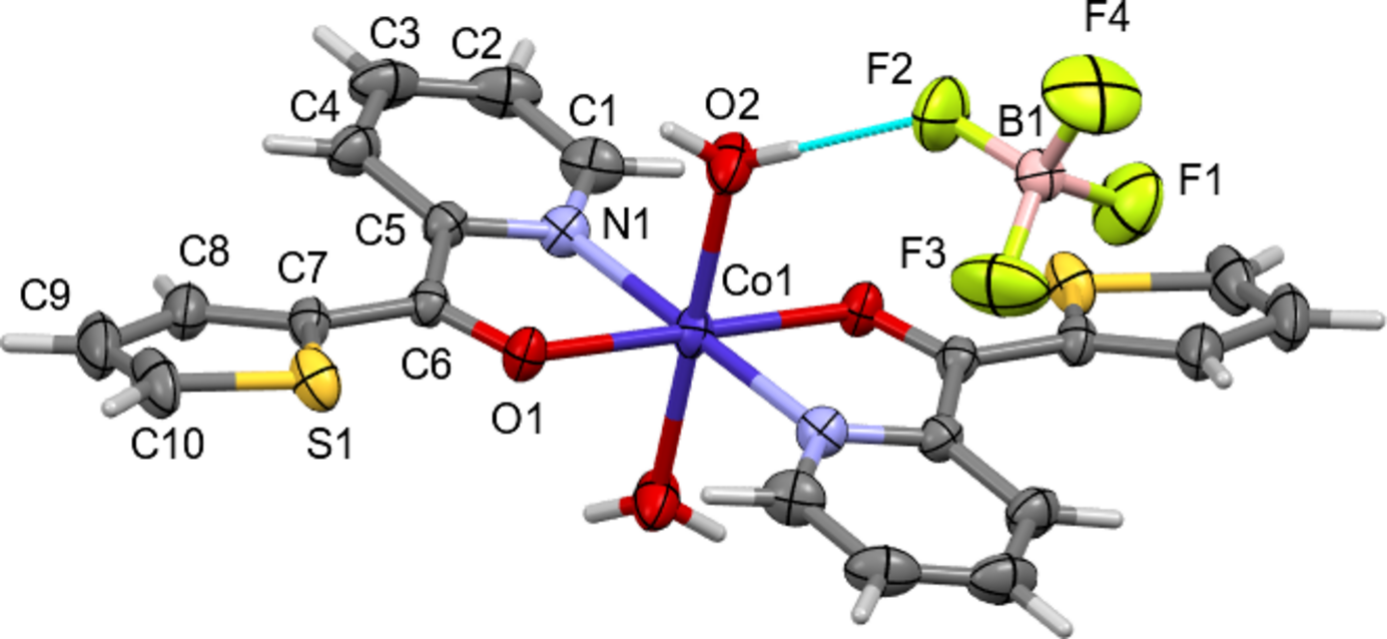
The mol­ecular entities in the crystal structure of the title salt shown with displacement ellipsoids at the 50% probability level. Atoms of the asymmetric unit are labeled and the disordered thienyl ring is shown only for the major component.

The complex title salt (Fig. 1 ▸) is structurally similar to the previously reported Cu^II^ (Sommerer *et al.*, 1998 ▸) and Ni^II^ (Westcott & Nichol, 2026 ▸) analogs. The Co^II^ site is located at an inversion center with pseudo-octa­hedral coordination. The 2-thienyl-2-pyridyl (tpk) ligands coordinate through pyridyl N [2.0189 (13) Å] and carbonyl O [2.0869 (11) Å] atoms in the equatorial plane, and oxygen atoms from water mol­ecules coordinate in the axial positions, leading to an [O_4_N_2_] coordination set. The Co—O distances with the aqua ligands are significantly shorter than in the Cu^II^ complex [2.0903 (13) Å *versus* 2.409 (3) Å], likely due to the Jahn–Teller distortion seen for octa­hedral Cu^II^ complexes (Procter *et al.*, 1968 ▸).

All other bond lengths and angles are consistent with the previously reported Cu^II^ (Sommerer *et al.*, 1998 ▸) and Ni^II^ (Westcott & Nichol, 2026 ▸) complexes, and with other Co^II^ complexes with similar ligands, such as di-2-pyridyl ketone (Crundwell *et al.*, 2003 ▸) or di-2-pyridyl ketone oxime (Stamou *et al.*, 2025 ▸).

**Figure 2:**
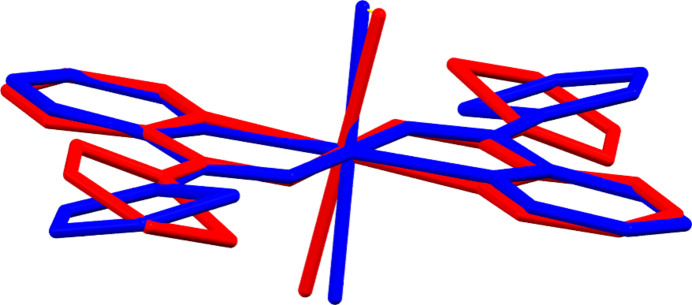
Overlay of the complex cation of the title compound (blue) with the previously reported Ni^II^ complex (red) showing the different orientations of the thienyl moiety.

The thienyl ring in the title complex is 21.22 (8)° out of the plane defined by the central metal cation and the pyridyl ring, while this value is 19.01 (7)° for the Ni^II^ complex (Westcott & Nichol 2026 ▸); however, the rotations are in opposite directions, leading to a difference of ∼40° (Fig. 2 ▸).

**Figure 3:**
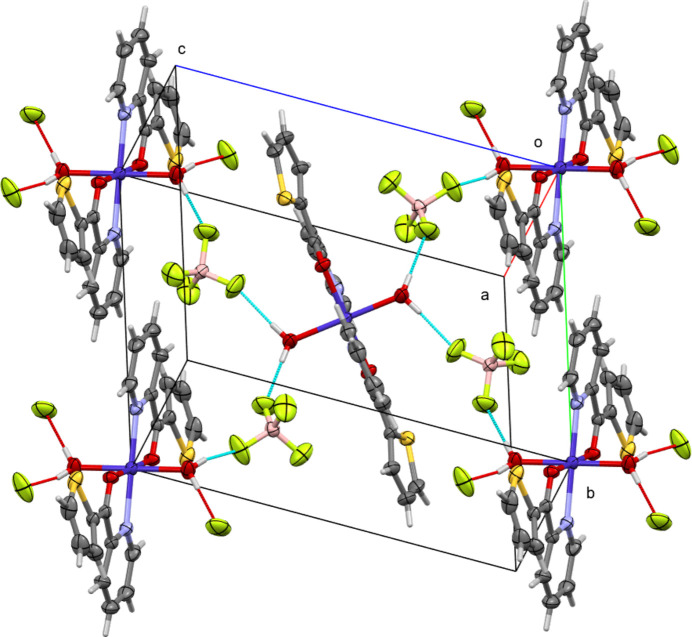
O—H⋯F hydrogen-bonding inter­actions (dashed lines) in the title compound showing a layered arrangement parallel to (10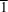).

The BF_4_^−^ anion acts as a hydrogen-bonding acceptor with the coordinating water mol­ecules as donors (Table 1 ▸), leading to a hydrogen-bonded layer extending parallel to (10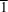), Fig. 3 ▸. Additional weak C—H⋯F inter­actions between the thio­phene moiety and the anion (Table 1 ▸) consolidate the packing.

## Synthesis and crystallization

Co(BF_4_)_2_·6H_2_O and aceto­nitrile were used as received from Thermo-Fisher; 2-thienyl 2-pyridyl ketone (tpk) was used as received from Rieke Metals. The title compound was synthesized following a literature procedure (Sommerer *et al.*, 1998 ▸): excess Co(BF_4_)_2_·6H_2_O (0.2108 g, 0.620 mmol) was combined with tpk (0.1825 g, 1.00 mmol) in 35 ml of aceto­nitrile at room temperature affording a dark-red solution, which was allowed to slowly evaporate until production and isolation of dark-orange crystals suitable for X-ray diffraction (30 d).

## Refinement

Crystal data, data collection, and structure refinement details are summarized in Table 2 ▸. The unit cell metrically fits an ortho­rhom­bic *C*-centered cell, and the crystal under investigation was twinned by a 180° rotation around the reciprocal *c* axis. Refinement as a two-component twin with application of the TWIN transformation matrix [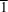 0 0/ 0 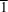 0/ 1 0 1] gave a BASF value of 0.2138 (8).

The thio­phene substituent is disordered by an approximate twofold rotation. The two disordered moieties were restrained to have similar geometries using the SAME restraint of *SHELXL* (Sheldrick, 2015*b* ▸). The minor moiety was restrained to be close to planar, and *U^ij^* components for disordered atoms closer to each other than 2.0 Å were restrained to be similar (SIMU 0.01 restraint). Subject to these conditions, the occupancy ratio refined to 0.951 (3):0.049 (3). Water H-atom positions were refined and O—H and H⋯H distances were restrained to 0.84 (2) and 1.37 (2) Å, respectively.

## Supplementary Material

Crystal structure: contains datablock(s) I. DOI: 10.1107/S2414314626007492/wm4252sup1.cif

Structure factors: contains datablock(s) I. DOI: 10.1107/S2414314626007492/wm4252Isup2.hkl

CCDC reference: 2574968

Additional supporting information:  crystallographic information; 3D view; checkCIF report

## Figures and Tables

**Table 1:** Hydrogen-bond geometry (Å, °)

*D*—H⋯*A*	*D*—H	H⋯*A*	*D*⋯*A*	*D*—H⋯*A*
O2—H2*A*⋯F4^i^	0.81 (1)	1.89 (1)	2.683 (2)	164 (3)
O2—H2*B*⋯F2	0.81 (1)	1.86 (1)	2.669 (2)	178 (4)
C2—H2⋯F1^ii^	0.95	2.52	3.352 (3)	147
C2—H2⋯F2^ii^	0.95	2.55	3.460 (3)	161
C4—H4⋯F1^iii^	0.95	2.57	3.207 (3)	124

Symmetry codes: (i) 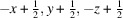; (ii) 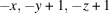; (iii) 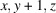.

**Table 2:** Experimental details

Crystal data	
Chemical formula	[Co(C_10_H_7_NOS)_3_(H_2_O)_2_](BF_4_)_2_
*M*_r_	647.03
Crystal system, space group	Monoclinic, *P*2_1_/*n*
Temperature (K)	150
*a*, *b*, *c* (Å)	7.2133 (4), 11.9888 (8), 15.2093 (10)
β (°)	103.623 (2)
*V* (Å^3^)	1278.28 (14)
*Z*	2
Radiation type	Mo *K*α
μ (mm^−1^)	0.93
Crystal size (mm)	0.24 × 0.21 × 0.15

Data collection	
Diffractometer	Bruker D8 Quest
Absorption correction	Multi-scan (*TWINABS*; Bruker, 2025 ▸)
*T*_min_, *T*_max_	0.618, 0.747
No. of measured, independent and observed [*I* > 2σ(*I*)] reflections	115558, 4990, 4783
*R*_int_	0.064
(sin θ/λ)_max_ (Å^−1^)	0.770

Refinement	
*R*[*F*^2^ > 2σ(*F*^2^)], *wR*(*F*^2^), *S*	0.034, 0.099, 1.05
No. of reflections	4990
No. of parameters	231
No. of restraints	168
H-atom treatment	H atoms treated by a mixture of independent and constrained refinement
Δρ_max_, Δρ_min_ (e Å^−3^)	0.92, −0.58

Computer programs: *APEX6* (Bruker, 2026 ▸), *SAINT* (Bruker, 2025 ▸), *SHELXT* (Sheldrick, 2015*a* ▸), *SHELXL* (Sheldrick, 2015*b* ▸), *ShelXle* (Hübschle *et al.*, 2011 ▸), *Mercury* (Macrae *et al.*, 2020 ▸) and *publCIF* (Westrip, 2010 ▸).

## full crystallographic data

### trans-Diaquabis(pyridin-2-yl thiophen-2-yl ketone-κ2N,O)cobalt(II) bis(tetrafluoridoborate) Crystal data

**Table:**

[Co(C_10_H_7_NOS)_3_(H_2_O)_2_](BF_4_)_2_	*F*(000) = 650
*M**_r_* = 647.03	*D*_x_ = 1.681 Mg m^−^^3^
Monoclinic, *P*2_1_/*n*	Mo *K*α radiation, λ = 0.71073 Å
*a* = 7.2133 (4) Å	Cell parameters from 9155 reflections
*b* = 11.9888 (8) Å	θ = 2.2–33.1°
*c* = 15.2093 (10) Å	µ = 0.93 mm^−^^1^
β = 103.623 (2)°	*T* = 150 K
*V* = 1278.28 (14) Å^3^	Block, orange
*Z* = 2	0.24 × 0.21 × 0.15 mm

### trans-Diaquabis(pyridin-2-yl thiophen-2-yl ketone-κ2N,O)cobalt(II) bis(tetrafluoridoborate) Data collection

**Table:**

Bruker D8 Quest diffractometer	4990 independent reflections
Radiation source: fine focus sealed tube X-ray source	4783 reflections with *I* > 2σ(*I*)
Triumph curved graphite crystal monochromator	*R*_int_ = 0.064
Detector resolution: 7.4074 pixels mm^-1^	θ_max_ = 33.2°, θ_min_ = 2.8°
ω and phi scans	*h* = −11→11
Absorption correction: multi-scan (*TWINABS*; Bruker, 2025)	*k* = −18→18
*T*_min_ = 0.618, *T*_max_ = 0.747	*l* = −23→23
115558 measured reflections	

### trans-Diaquabis(pyridin-2-yl thiophen-2-yl ketone-κ2N,O)cobalt(II) bis(tetrafluoridoborate) Refinement

**Table:**

Refinement on *F*^2^	Primary atom site location: dual
Least-squares matrix: full	Secondary atom site location: difference Fourier map
*R*[*F*^2^ > 2σ(*F*^2^)] = 0.034	Hydrogen site location: mixed
*wR*(*F*^2^) = 0.099	H atoms treated by a mixture of independent and constrained refinement
*S* = 1.05	*w* = 1/[σ^2^(*F*_o_^2^) + (0.059*P*)^2^ + 0.4235*P*] where *P* = (*F*_o_^2^ + 2*F*_c_^2^)/3
4990 reflections	(Δ/σ)_max_ = 0.001
231 parameters	Δρ_max_ = 0.92 e Å^−^^3^
168 restraints	Δρ_min_ = −0.58 e Å^−^^3^

### trans-Diaquabis(pyridin-2-yl thiophen-2-yl ketone-κ2N,O)cobalt(II) bis(tetrafluoridoborate) Special details

**Table:**

Geometry. All esds (except the esd in the dihedral angle between two l.s. planes) are estimated using the full covariance matrix. The cell esds are taken into account individually in the estimation of esds in distances, angles and torsion angles; correlations between esds in cell parameters are only used when they are defined by crystal symmetry. An approximate (isotropic) treatment of cell esds is used for estimating esds involving l.s. planes.
Refinement. The unit cell does metrically fit an orthorhombic C-centered cell and is twinned by this symmetry (180 degree rotation around reciprocal c (0 0 1). Application of the TWIN transformation matrix -1 0 0 0 -1 0 1 0 1 gave a BASF value of 0.2138 (8).Refined as a 2-component twin.The thiophene substituent is disordered by an approximate two-fold rotation. The two disordered moieties were restrained to have similar geometries (SAME restraint of ShelXL). The minor moiety was restrained to be close to planar. Uij components of ADPs for disordered atoms closer to each other than 2.0 Angstrom were restrained to be similar (SIMU 0.01 restraint). Subject to these conditions the occupancy ratio refined to 0.951 (3) to 0.049 (3). Water H atom positions were refined and O-H and H···H distances were restrained to 0.84 (2) and 1.37 (2) Angstrom, respectively. Some water H atom positions were further restrained based on hydrogen bonding considerations.

### trans-Diaquabis(pyridin-2-yl thiophen-2-yl ketone-κ2N,O)cobalt(II) bis(tetrafluoridoborate) Fractional atomic coordinates and isotropic or equivalent isotropic displacement parameters (Å^2^)

**Table:**

	*x*	*y*	*z*	*U*_iso_*/*U*_eq_	Occ. (<1)
Co1	0.500000	0.500000	0.500000	0.02485 (7)	
F1	0.2496 (3)	0.09814 (16)	0.40896 (11)	0.0684 (5)	
F2	0.2007 (3)	0.25203 (12)	0.32495 (14)	0.0682 (5)	
F3	0.4698 (2)	0.1494 (2)	0.33606 (13)	0.0761 (6)	
F4	0.1826 (3)	0.08622 (19)	0.25758 (13)	0.0830 (6)	
N1	0.35744 (18)	0.65047 (11)	0.50282 (9)	0.0272 (2)	
C1	0.2108 (3)	0.66595 (17)	0.54098 (12)	0.0357 (3)	
H1	0.172569	0.606240	0.573802	0.043*	
O1	0.63194 (17)	0.60567 (9)	0.42424 (8)	0.0278 (2)	
O2	0.2908 (2)	0.45919 (11)	0.38414 (9)	0.0413 (3)	
H2A	0.279 (5)	0.503 (2)	0.3424 (15)	0.062*	
H2B	0.266 (5)	0.3962 (13)	0.3655 (19)	0.062*	
C2	0.1118 (3)	0.76632 (19)	0.53445 (13)	0.0422 (4)	
H2	0.010753	0.775885	0.564101	0.051*	
C3	0.1625 (3)	0.85102 (18)	0.48457 (14)	0.0434 (4)	
H3	0.096248	0.920120	0.478913	0.052*	
C4	0.3128 (3)	0.83522 (14)	0.44185 (12)	0.0354 (3)	
H4	0.347505	0.892141	0.405316	0.043*	
C5	0.4095 (2)	0.73449 (12)	0.45430 (9)	0.0252 (2)	
C6	0.5748 (2)	0.70440 (11)	0.41513 (9)	0.0238 (2)	
S1	0.81191 (9)	0.72350 (5)	0.30243 (3)	0.03521 (14)	0.951 (3)
C7	0.6698 (3)	0.78152 (15)	0.36741 (12)	0.0267 (3)	0.951 (3)
C8	0.6793 (5)	0.8966 (3)	0.3668 (2)	0.0385 (6)	0.951 (3)
H8	0.613994	0.943791	0.399664	0.046*	0.951 (3)
C9	0.7978 (4)	0.9359 (2)	0.31144 (17)	0.0475 (5)	0.951 (3)
H9	0.819995	1.012702	0.302468	0.057*	0.951 (3)
C10	0.8764 (4)	0.8518 (2)	0.27245 (15)	0.0457 (5)	0.951 (3)
H10	0.958393	0.863475	0.232761	0.055*	0.951 (3)
S1B	0.641 (3)	0.9146 (15)	0.3538 (13)	0.040 (2)	0.049 (3)
C7B	0.662 (5)	0.772 (2)	0.365 (2)	0.036 (3)	0.049 (3)
C8B	0.788 (6)	0.731 (3)	0.316 (3)	0.037 (3)	0.049 (3)
H8B	0.816978	0.653858	0.312593	0.044*	0.049 (3)
C9B	0.867 (6)	0.816 (3)	0.273 (3)	0.041 (3)	0.049 (3)
H9B	0.958416	0.803783	0.238039	0.050*	0.049 (3)
C10B	0.797 (5)	0.918 (3)	0.286 (2)	0.041 (3)	0.049 (3)
H10B	0.831310	0.983895	0.259243	0.049*	0.049 (3)
B1	0.2782 (3)	0.14565 (15)	0.33038 (12)	0.0294 (3)	

### trans-Diaquabis(pyridin-2-yl thiophen-2-yl ketone-κ2N,O)cobalt(II) bis(tetrafluoridoborate) Atomic displacement parameters (Å^2^)

**Table:**

	*U*^11^	*U*^22^	*U*^33^	*U*^12^	*U*^13^	*U*^23^
Co1	0.03299 (13)	0.02138 (11)	0.02170 (11)	−0.00081 (9)	0.00948 (10)	0.00206 (9)
F1	0.0812 (12)	0.0736 (11)	0.0577 (9)	0.0076 (9)	0.0306 (8)	0.0260 (8)
F2	0.0762 (11)	0.0261 (5)	0.0926 (12)	0.0049 (6)	0.0007 (10)	0.0003 (7)
F3	0.0369 (7)	0.1274 (17)	0.0669 (10)	0.0009 (9)	0.0183 (7)	0.0025 (11)
F4	0.0865 (13)	0.0901 (14)	0.0624 (9)	−0.0011 (11)	−0.0025 (9)	−0.0491 (10)
N1	0.0301 (5)	0.0292 (5)	0.0239 (5)	0.0016 (4)	0.0095 (4)	−0.0016 (4)
C1	0.0336 (7)	0.0469 (9)	0.0293 (6)	0.0015 (6)	0.0127 (6)	−0.0051 (6)
O1	0.0345 (5)	0.0221 (4)	0.0295 (5)	0.0034 (4)	0.0131 (4)	0.0039 (3)
O2	0.0612 (8)	0.0278 (5)	0.0286 (5)	−0.0070 (6)	−0.0018 (5)	0.0033 (4)
C2	0.0302 (7)	0.0576 (12)	0.0388 (8)	0.0089 (7)	0.0082 (6)	−0.0153 (8)
C3	0.0336 (7)	0.0439 (9)	0.0490 (10)	0.0135 (7)	0.0022 (7)	−0.0126 (8)
C4	0.0358 (7)	0.0284 (7)	0.0395 (8)	0.0078 (6)	0.0037 (6)	−0.0016 (6)
C5	0.0278 (6)	0.0232 (5)	0.0239 (5)	0.0018 (5)	0.0046 (4)	−0.0017 (4)
C6	0.0284 (6)	0.0221 (5)	0.0207 (5)	−0.0007 (4)	0.0050 (4)	0.0008 (4)
S1	0.0430 (3)	0.0378 (2)	0.0284 (2)	−0.01227 (17)	0.01575 (17)	−0.00512 (16)
C7	0.0345 (7)	0.0227 (6)	0.0224 (6)	−0.0030 (5)	0.0059 (5)	0.0016 (5)
C8	0.0442 (14)	0.0280 (11)	0.0403 (13)	−0.0046 (9)	0.0037 (10)	0.0059 (8)
C9	0.0605 (12)	0.0359 (9)	0.0427 (11)	−0.0152 (9)	0.0054 (10)	0.0131 (8)
C10	0.0578 (12)	0.0489 (12)	0.0317 (8)	−0.0223 (10)	0.0132 (8)	0.0083 (8)
S1B	0.048 (4)	0.032 (4)	0.038 (4)	−0.009 (4)	0.005 (4)	0.002 (4)
C7B	0.043 (5)	0.030 (5)	0.033 (5)	−0.006 (5)	0.006 (5)	−0.002 (5)
C8B	0.046 (5)	0.036 (5)	0.029 (5)	−0.014 (5)	0.010 (5)	0.000 (5)
C9B	0.051 (5)	0.039 (5)	0.033 (5)	−0.014 (5)	0.008 (5)	0.003 (5)
C10B	0.050 (5)	0.036 (5)	0.035 (5)	−0.014 (5)	0.009 (5)	0.008 (5)
B1	0.0329 (7)	0.0273 (7)	0.0283 (7)	−0.0003 (6)	0.0080 (6)	−0.0047 (5)

### trans-Diaquabis(pyridin-2-yl thiophen-2-yl ketone-κ2N,O)cobalt(II) bis(tetrafluoridoborate) Geometric parameters (Å, º)

**Table:**

Co1—N1	2.0819 (13)	C4—C5	1.385 (2)
Co1—N1^i^	2.0819 (13)	C4—H4	0.9500
Co1—O1	2.0869 (11)	C5—C6	1.497 (2)
Co1—O1^i^	2.0869 (11)	C6—C7B	1.36 (3)
Co1—O2	2.0902 (13)	C6—C7	1.444 (2)
Co1—O2^i^	2.0903 (13)	S1—C10	1.700 (2)
F1—B1	1.383 (2)	S1—C7	1.729 (2)
F2—B1	1.387 (2)	C7—C8	1.382 (4)
F3—B1	1.365 (2)	C8—C9	1.414 (4)
F4—B1	1.360 (2)	C8—H8	0.9500
N1—C1	1.334 (2)	C9—C10	1.359 (4)
N1—C5	1.3531 (19)	C9—H9	0.9500
C1—C2	1.391 (3)	C10—H10	0.9500
C1—H1	0.9500	S1B—C10B	1.698 (19)
O1—C6	1.2502 (17)	S1B—C7B	1.73 (2)
O2—H2A	0.811 (13)	C7B—C8B	1.39 (2)
O2—H2B	0.812 (13)	C8B—C9B	1.413 (19)
C2—C3	1.368 (3)	C8B—H8B	0.9500
C2—H2	0.9500	C9B—C10B	1.345 (19)
C3—C4	1.402 (3)	C9B—H9B	0.9500
C3—H3	0.9500	C10B—H10B	0.9500
			
N1—Co1—N1^i^	180.0	O1—C6—C7B	116.0 (15)
N1—Co1—O1	77.12 (5)	O1—C6—C7	118.63 (14)
N1^i^—Co1—O1	102.88 (5)	O1—C6—C5	117.16 (12)
N1—Co1—O1^i^	102.88 (5)	C7B—C6—C5	126.7 (15)
N1^i^—Co1—O1^i^	77.12 (5)	C7—C6—C5	124.21 (14)
O1—Co1—O1^i^	180.0	C10—S1—C7	91.47 (11)
N1—Co1—O2	87.60 (6)	C8—C7—C6	132.3 (2)
N1^i^—Co1—O2	92.40 (6)	C8—C7—S1	111.14 (19)
O1—Co1—O2	90.53 (5)	C6—C7—S1	116.43 (13)
O1^i^—Co1—O2	89.48 (5)	C7—C8—C9	112.0 (3)
N1—Co1—O2^i^	92.40 (6)	C7—C8—H8	124.0
N1^i^—Co1—O2^i^	87.60 (6)	C9—C8—H8	124.0
O1—Co1—O2^i^	89.47 (5)	C10—C9—C8	112.7 (2)
O1^i^—Co1—O2^i^	90.52 (5)	C10—C9—H9	123.7
O2—Co1—O2^i^	180.00 (5)	C8—C9—H9	123.7
C1—N1—C5	118.95 (15)	C9—C10—S1	112.68 (17)
C1—N1—Co1	125.15 (12)	C9—C10—H10	123.7
C5—N1—Co1	115.59 (9)	S1—C10—H10	123.7
N1—C1—C2	122.40 (18)	C10B—S1B—C7B	91.6 (13)
N1—C1—H1	118.8	C6—C7B—C8B	122 (3)
C2—C1—H1	118.8	C6—C7B—S1B	127 (3)
C6—O1—Co1	117.04 (9)	C8B—C7B—S1B	110.4 (17)
Co1—O2—H2A	115 (2)	C7B—C8B—C9B	112 (2)
Co1—O2—H2B	125 (2)	C7B—C8B—H8B	123.8
H2A—O2—H2B	111 (2)	C9B—C8B—H8B	123.8
C3—C2—C1	118.82 (17)	C10B—C9B—C8B	112 (2)
C3—C2—H2	120.6	C10B—C9B—H9B	123.8
C1—C2—H2	120.6	C8B—C9B—H9B	123.8
C2—C3—C4	119.58 (17)	C9B—C10B—S1B	113.2 (18)
C2—C3—H3	120.2	C9B—C10B—H10B	123.4
C4—C3—H3	120.2	S1B—C10B—H10B	123.4
C5—C4—C3	118.29 (18)	F4—B1—F3	112.37 (18)
C5—C4—H4	120.9	F4—B1—F1	109.74 (19)
C3—C4—H4	120.9	F3—B1—F1	108.29 (17)
N1—C5—C4	121.89 (15)	F4—B1—F2	108.26 (18)
N1—C5—C6	112.77 (12)	F3—B1—F2	111.16 (19)
C4—C5—C6	125.32 (14)	F1—B1—F2	106.87 (17)
			
C5—N1—C1—C2	−1.5 (2)	C5—C6—C7—C8	21.1 (3)
Co1—N1—C1—C2	−174.79 (14)	O1—C6—C7—S1	16.1 (2)
N1—C1—C2—C3	2.2 (3)	C5—C6—C7—S1	−163.28 (11)
C1—C2—C3—C4	−0.4 (3)	C10—S1—C7—C8	−1.8 (2)
C2—C3—C4—C5	−2.1 (3)	C10—S1—C7—C6	−178.32 (15)
C1—N1—C5—C4	−1.1 (2)	C6—C7—C8—C9	177.5 (2)
Co1—N1—C5—C4	172.80 (12)	S1—C7—C8—C9	1.7 (3)
C1—N1—C5—C6	−179.52 (13)	C7—C8—C9—C10	−0.7 (3)
Co1—N1—C5—C6	−5.59 (15)	C8—C9—C10—S1	−0.7 (3)
C3—C4—C5—N1	2.9 (2)	C7—S1—C10—C9	1.4 (2)
C3—C4—C5—C6	−178.94 (15)	O1—C6—C7B—C8B	9.1 (17)
Co1—O1—C6—C7B	−179.8 (12)	C5—C6—C7B—C8B	−166.4 (15)
Co1—O1—C6—C7	176.64 (11)	O1—C6—C7B—S1B	−170.0 (15)
Co1—O1—C6—C5	−3.91 (16)	C5—C6—C7B—S1B	15 (3)
N1—C5—C6—O1	6.33 (18)	C10B—S1B—C7B—C6	179 (2)
C4—C5—C6—O1	−171.99 (15)	C10B—S1B—C7B—C8B	−0.2 (13)
N1—C5—C6—C7B	−178.3 (14)	C6—C7B—C8B—C9B	−178 (3)
C4—C5—C6—C7B	3.4 (14)	S1B—C7B—C8B—C9B	1.4 (18)
N1—C5—C6—C7	−174.25 (14)	C7B—C8B—C9B—C10B	−2 (3)
C4—C5—C6—C7	7.4 (2)	C8B—C9B—C10B—S1B	2 (4)
O1—C6—C7—C8	−159.5 (3)	C7B—S1B—C10B—C9B	−1 (3)

Symmetry code: (i) −*x*+1, −*y*+1, −*z*+1.

### trans-Diaquabis(pyridin-2-yl thiophen-2-yl ketone-κ2N,O)cobalt(II) bis(tetrafluoridoborate) Hydrogen-bond geometry (Å, º)

**Table:**

*D*—H···*A*	*D*—H	H···*A*	*D*···*A*	*D*—H···*A*
O2—H2*A*···F4^ii^	0.81 (1)	1.89 (1)	2.683 (2)	164 (3)
O2—H2*B*···F2	0.81 (1)	1.86 (1)	2.669 (2)	178 (4)
C2—H2···F1^iii^	0.95	2.52	3.352 (3)	147
C2—H2···F2^iii^	0.95	2.55	3.460 (3)	161
C4—H4···F1^iv^	0.95	2.57	3.207 (3)	124

Symmetry codes: (ii) −*x*+1/2, *y*+1/2, −*z*+1/2; (iii) −*x*, −*y*+1, −*z*+1; (iv) *x*, *y*+1, *z*.
